# Neurovascular Contacts in the Pathophysiology of Neuralgic Amyotrophy: An Observational Study

**DOI:** 10.1002/acn3.70416

**Published:** 2026-04-25

**Authors:** Johannes Fabian Holle, Andreas Leha, Volker Limmroth, Wolfram Windisch, Maximilian Zimmermann

**Affiliations:** ^1^ Health Faculty/Department for Human Medicine University of Witten/Herdecke Germany; ^2^ Department of Neurology, Cologne‐Merheim Hospitals of the City of Cologne Cologne Germany; ^3^ Department of Medical Statistics University Medical Center Göttingen Germany; ^4^ Cologne‐Merheim Lung Clinic Hospitals of the City of Cologne Cologne Germany

**Keywords:** neuralgic amyotrophy, neurovascular contacts, Parsonage–Turner syndrome, pathophysiology

## Abstract

**Objective:**

Neuralgic amyotrophy (NA) is a prevalent, monophasic, multifocal immune‐mediated neuropathy. A distinctive characteristic of the disease is the occurrence of nerve or fascicle constrictions and torsions (NA‐associated focal nerve lesions, NAFL). The pathophysiology underlying this phenomenon remains to be fully elucidated.

**Methods:**

This study uses an observational research design with a single center, retrospective approach. We evaluated patients who presented at the outpatient clinic of a tertiary referral hospital between January 1, 2022, and August 5, 2025, due to NA. In addition to evaluating clinical and neurophysiological data, two independent experts examined the available ultrasound images for the prevalence of NAFL and the presence of arterial vascular structures in the immediate vicinity.

**Results:**

A total of 171 affected nerves from 77 patients were analyzed. The prevalence of NAFL was 31% (24/77) among all patients. In 53% of all NAFL, direct neurovascular contact could be detected. A previous mechanical trigger was identified in 71% of patients with proven NAFL but without neurovascular contact. This was the case in only 29% of patients with neurovascular contact, although this discrepancy did not attain statistical significance.

**Interpretation:**

The frequency of neurovascular contacts in close proximity to NAFL suggests that these contacts play a role in its pathophysiology. Such contacts, similar to mechanical tension on the nerve, could lead to a local disruption of the blood–nerve barrier. This would explain phenomena inconsistent with the current pathophysiological model, such as the lack of preference for the dominant arm and NAFL's occurrence at certain anatomical sites.

## 1 Introduction

According to the current understanding, neuralgic amyotrophy (NA) is a multifactorial disease with a complex pathophysiology that typically results in mostly monophasic and often multifocal neuritis [1].

A substantial body of evidence supports the hypothesis that the pathophysiology of NA is characterized by a cascade‐like sequence of different processes. The cascade is likely initiated by an immunostimulatory factor, such as hepatitis E infection, which results in the development of autoreactive B and/or T lymphocytes. Typically, the integrity of the blood–nerve barrier serves as a barrier to the migration of these cells into the endoneural space. In accordance with the extant literature, the occurrence of microtrauma resulting from movement‐ and position‐related tensile stress on the nerve constitutes an additional necessary factor. This results in a transient increase in the permeability of the blood–nerve barrier, thereby enabling autoreactive immune cells to initiate an inflammatory process within the endoneural space [2, 3]. The anatomy of the shoulder joint allows for a wide range of motion. This exposes the proximal nerves of the upper extremities to considerable tensile stress in everyday life and could explain why NA occurs more frequently in this region of the body [2, 4].

A specific phenomenon of neuralgic amyotrophy is the formation of constrictions and torsions in the affected nerves or individual fascicles. Nerve constriction refers to the fibrosis and adhesions of the epineurium and perineurium caused by the inflammatory process. Conversely, torsions are defined as the twisting of a nerve or its individual fascicles, likely occurring under the influence of voluntary limb movements on inflamed nerve segments [5]. In both cases, ultrasound imaging reveals a focal, hourglass‐like narrowing of the nerve lumen with abrupt changes in diameter. Although a complete narrowing of the nerve diameter, pronounced swelling proximal to the narrowing, and fascicle entwinement, in which fascicles twist against each other, are indicative of torsion, distinguishing between these two pathologies solely based on image morphology is challenging [6]. Due to the aforementioned reasons, the two entities are grouped together under the term “NA‐associated focal nerve lesions (NAFL).”

Nevertheless, in light of this model, certain observations derived from preceding studies remain to be fully elucidated.

1. For nearly all nerves affected by NA, no preference for the dominant arm can be determined, despite the greater mechanical stress expected there.
2. Most affected nerves have anatomical predilection sites that often do not correspond to locations with the greatest movement‐related mechanical stress.
3. Certain predilection sites can also be identified within the nerve cross‐section. In the case of the median nerve, for instance, the dorsal and dorsomedial fascicles are primarily affected [7].

Therefore, it can be assumed that, in addition to the postulated movement‐ and position‐dependent tensile stresses, other mechanical factors can explain the aforementioned observations. During routine nerve sonography of individuals with NA, the study's authors found that arterial vascular structures are often located near typical hourglass‐like nerve constrictions. Our work aimed to determine the prevalence of such contacts. The initial hypothesis was that this local coincidence occurs more frequently than expected by chance and is a pathogenetic factor in the development of such NA‐associated focal nerve lesions.

## 2 Methods

Data collection was performed at a single center and was retrospective. The study included patients who presented to the neurology outpatient clinic of a tertiary referral hospital between January 1, 2022, and August 5, 2025, due to NA. NA was diagnosed based on the clinical criteria published by van Alfen et al. in 2008 [8].

We recorded demographic data such as age and sex, as well as clinical information such as the location and severity of the initial pain syndrome, the latency period until the onset of paresis, and any previous immunological or mechanical trigger factors (primarily, activities of an athletic or occupational nature that result in excessive strain on the muscles of the shoulder girdle beyond the levels experienced in the course of everyday life). All patients underwent electromyography of the affected muscles. Absence of voluntary activity, a high‐frequency single discharge pattern, pathological spontaneous activity, and/or neurogenic changes (a rate of polyphasic and/or high‐amplitude motor unit potentials of > 30%) were considered evidence of nerve involvement. The involvement of the phrenic nerve was confirmed by ultrasound examination of the diaphragm when either a paradoxical upward movement or a lack of increase in thickness during inspiration was documented. Involvement of the recurrent laryngeal nerve was confirmed by indirect laryngoscopy. Thirty‐one patients underwent diaphragmatic sonography regardless of their medical history. The remaining patients only underwent the procedure if there were indications of diaphragmatic involvement (61/77 of all patients).

**FIGURE 1:**
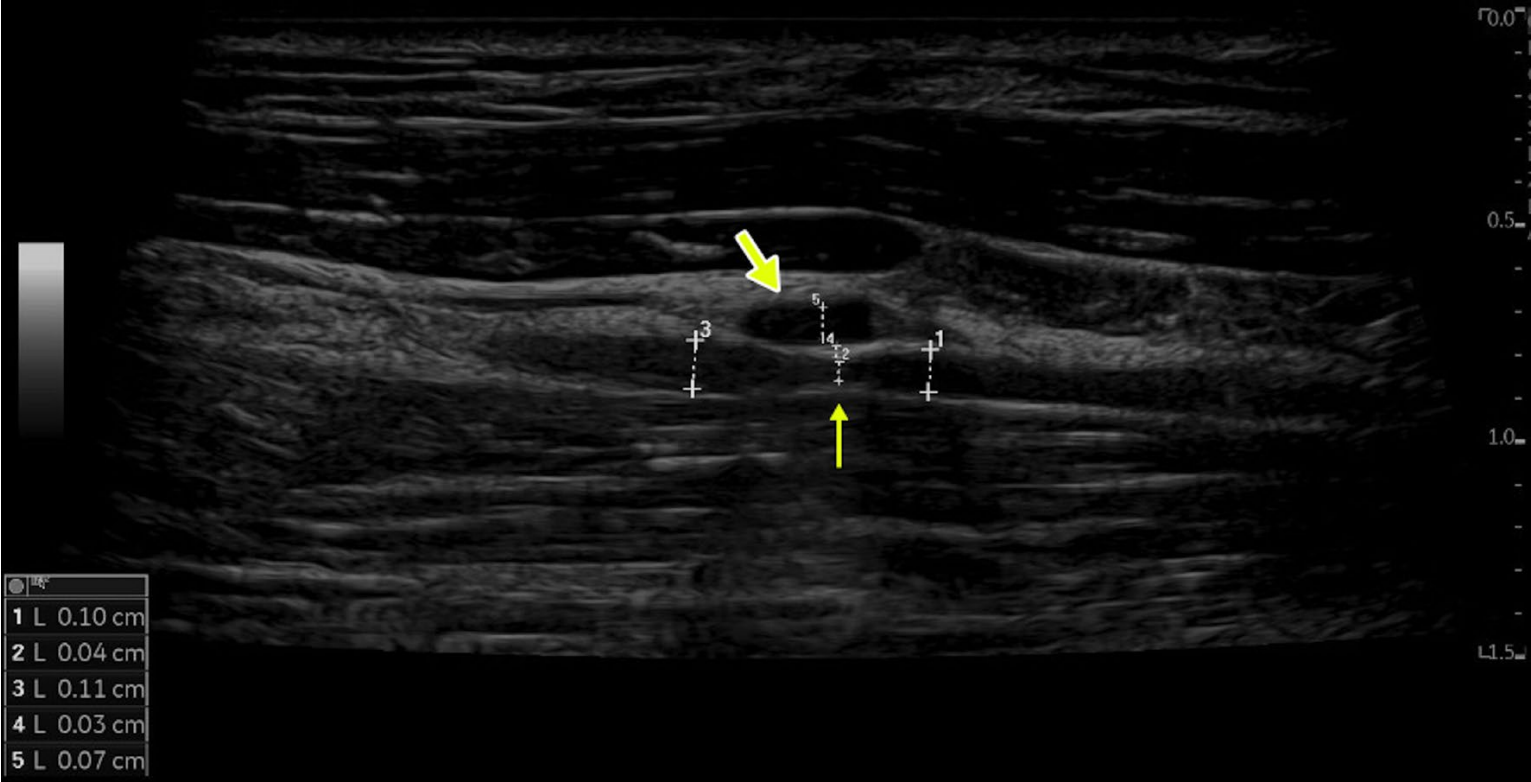
Neurovascular contact between a NAFL of the phrenic nerve (small arrow) and the transverse cervical artery (large arrow).

**FIGURE 2:**
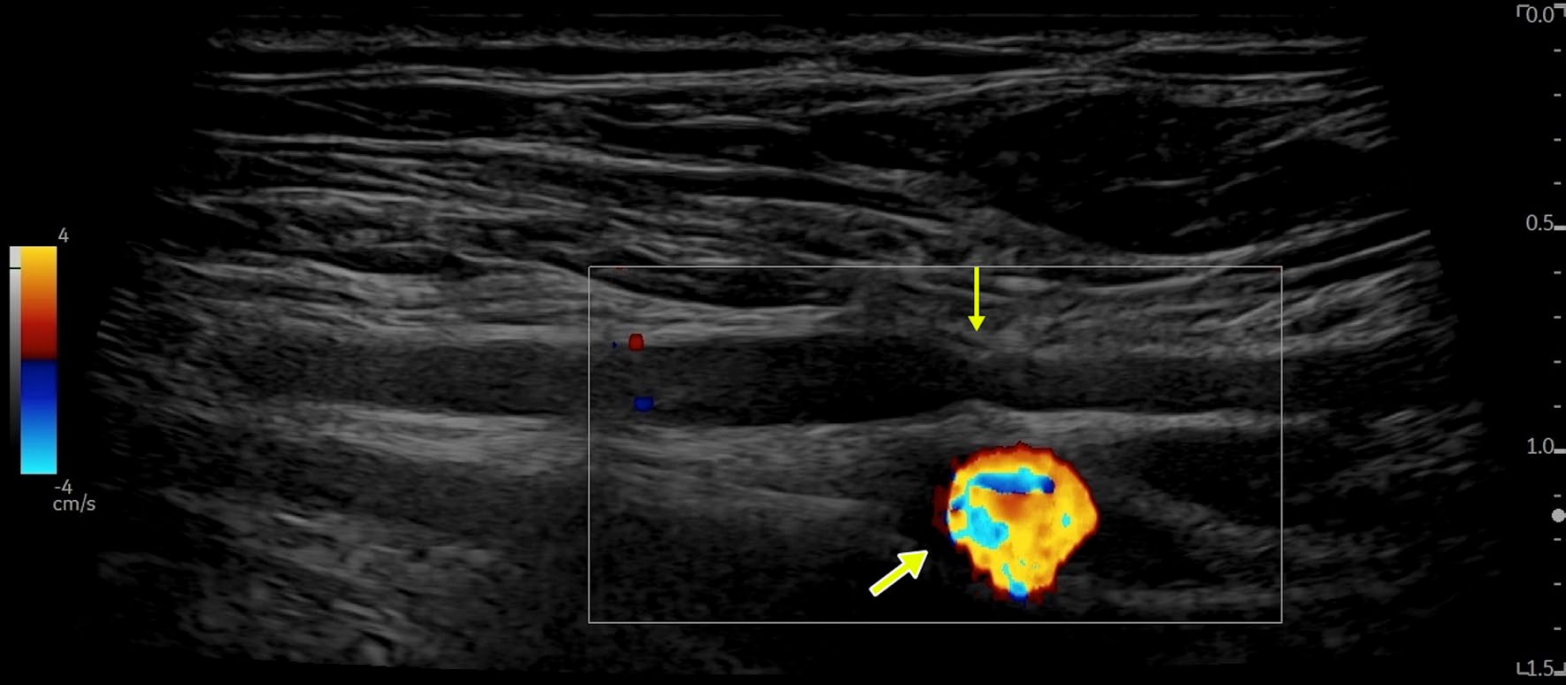
Neurovascular contact between a NAFL of the suprascapular nerve (small arrow) and the dorsal scapular artery (large arrow).

Where anatomically possible, all affected nerves were examined via high‐resolution sonography. This was performed using either a Philips Epiq 5 ultrasound system with an 18 MHz linear transducer or a GE Healthcare Logiq Fortis ultrasound system with a 24 MHz linear transducer. According to the literature, a NAFL was defined as an hourglass‐shaped focal indentation of a nerve or fascicle in the short and long axes, accompanied by cross‐sectional enlargement proximal and distal to the indentation [9]. Multiple images of all nerves examined by sonography were available along the longitudinal axis. These images were examined for arterial vessel structures crossing transversely to the nerve course in the immediate vicinity of the NAFL. We defined the term “immediate vicinity” as a distance of less than three millimeters between the outer vessel lumen and the maximum constriction (Figures 1 and 2). As there was a lack of available data for NA, the most comparable pathophysiological model of trigeminal neuralgia was used to define this condition. Radiological studies using high‐resolution MRI showed that a distance of ≤ 3 mm was strongly associated with symptom occurrence [10]. Arterial vascular structures were identified sonographically based on their nearly circular cross‐sectional shape and clear visualization of the intima‐media complex as a two‐layer, echogenic line. In color duplex sonography, the vessels had to exhibit a pulsating, directional color signal with laminar flow characteristics in pulsed‐wave (PW) Doppler sonography. Two experienced examiners identified the arteries independently.

Serological findings for hepatitis E IgG and IgM antibodies were available for 58 patients. Thirty of these patients were participants in a previous prospective study aimed at identifying various antibodies/antibody panels for the early diagnosis of NA (the “DANA Trial”: Development of an Assay for the Diagnosis of Neuralgic Amyotrophy). Inclusion criteria were a diagnosis of NA and symptom onset within the previous 8 weeks. Serological testing had been performed on the remaining patients as part of their previous outpatient evaluation before they were referred to our clinic.

The data were descriptively summarized by mean and standard deviations or by median with lower and upper quartile or by absolute and relative frequencies, as appropriate. Two‐group comparisons were done using *t*‐tests, Boschloo's exact tests, or Brunner‐Munzel tests, as indicated. The significance level was set to alpha = 5% for all the statistical tests. All analyses were performed with the statistical software R (version 4.4.0; R Core Team [11]) using the R‐package emmeans (version 1.10.7; Lenth [12]) for the contrast tests. The graphics were created using Prism 10 for macOS Version 10.6.1 (799), September 8, 2025.

Approval to conduct this study was obtained from the Witten/Herdecke University Ethics Committee (application number S‐124/2025). Because the data were analyzed anonymously, patient consent was not deemed necessary.

## 3 Results

Data from a total of 77 patients could be evaluated, of whom 24 (31.2%) had at least one NAFL. Of the 171 affected nerves, 34 (19.9%) showed NAFL on ultrasound. The mean age of the entire cohort was 50 ± 13 years, and this value did not differ between the subgroups with and without NAFL (Tables 1 and 2). Conversely, the group with NAFL exhibited a significantly greater proportion of male patients (88% versus 66%; *p* = 0.049). Additionally, there were substantial differences in terms of initial pain intensity (9 (8–10) versus 8 (7–9); *p* = 0.030) and the number of nerves affected (2.5 (1.8–3.0) versus 2.0 (1.0–2.0); *p* = 0.016), with higher values observed in the group with NAFL in each case.

**TABLE 1:** Overall structure of the group, both as a whole and in subgroups with and without NAFL.

	Overall (*n* = 77)	NAFL (*n* = 24)	No NAFL (*n* = 53)	*p*
Age (y)	50 ± 13	53 ± 13	49 ± 12	*p* = 0.151^a^
Sex (male in %)	72.7	87.5	66.0	*p* = 0.049^b^
M:F	2.7: 1	7: 1	1.9: 1	
Delay until presentation (d)	68 (37–278)	140 (52–273)	61 (30–274)	*p* = 0.328^c^
Pain intensity (NRS)	8 (7–9)	9 (8–10)	8 (7–9)	*p* = 0.030^c^
Delay until paresis (d)	7.0 ± 12.9	8.3 ± 13.8	6.3 ± 12.6	*p* = 0.643^c^
Bilateral involvement (%)	32.5 (25/77)	45.8 (11/24)	26.4 (14/53)	*p* = 0.093^b^
Nerves affected (*n*)	2.0 (1.0–3.0)	2.5 (1.8–3.0)	2.0 (1.0–2.0)	*p* = 0.016^c^
Mononeuropathy (%)	39.0 (30/77)	25.0 (6/24)	45.3 (24/53)	*p* = 0.107^b^
Mechanical trigger (%)	41 (25/61)	42.9 (9/21)	40 (16/40)	*p* = 1.000^b^
Immunological trigger (%)	55.4 (36/65)	40 (8/20)	62.2 (28/45)	*p* = 0.187^b^
Hep E IgM (%)	22.4 (13/58)	46.7 (7/15)	14.0 (6/43)	*p* = 0.019^b^
Family history (%)	4.7 (3/64)	0 (0/22)	7.1 (3/42)	*p* = 0.472^b^
Recurrence (%)	14.9 (10/67)	13.6 (3/22)	15.6 (7/45)	*p* = 1.000^b^
Corticosteroid (%)	61.9 (39/63)	59.1 (13/22)	63.4 (26/41)	*p* = 0.765^b^
Neurovascular contact (%)	—	62.5 (15/24)	—	—

Abbreviations: d, days; F, female; M, male; NAFL, neuralgic amyotrophy‐associated focal nerve lesions; NRS, numeric ratings scale; y, years.

^a^ Welch two sample *t*‐test.

^b^ Boschloo's exact test.

^c^ Brunner‐Munzel test.

**TABLE 2:** Affected nerves.

	Affected nerves left	Affected nerves right	NAFL left	NAFL right	NAFL (% affected nerve)	Neurovascular contact left	Neurovascular contact right	Neurovascular contact (% NAFL)
PHR	25/56	31/56	8/14	6/14	25 (14/56)	4/5	1/5	35.7 (5/14)
THL	7/33	26/33	1/5	4/5	15.2 (5/33)	1/3	2/3	60 (3/5)
SUP	19/31	12/31	5/7	2/7	22.6 (7/31)	4/6	2/6	85.7 (6/7)
RAD	10/15	5/15	3/3	0/3	20 (3/15)	1/1	0/1	33.3 (1/3)
IOA	6/13	7/13	0	0	0	0	0	0
MED	6/8	2/8	1/4	3/4	19 (4/21)^a^	0/2	2/2	50 (2/4)
MUS	2/5	3/5	0/1	1/1	20 (1/5)	0/1	1/1	100 (1/1)
REC	1/3	2/3	0	0	0	0	0	0
ULN	3/3	0/0	0	0	0	0	0	0
IOP	2/2	0/0	0	0	0	0	0	0
AX	1/1	0/0	0	0	0	0	0	0
DSC	0/0	1/1	0	0	0	0	0	0
Total/mean	82/171	89/171	18/34	16/34	19.9 (34/171)	10/18	8/18	52.9 (18/34)

Abbreviations: AX, axillar nerve; DSC, dorsal scapular nerve; IOA, anterior interosseous nerve; IOP, posterior interosseous nerve; MED, median nerve; MUS, musculocutaneous nerve; NAFL, neuralgic amyotrophy‐associated focal nerve lesions; PHR, phrenic nerve; RAD, radial nerve; REC, recurrent nerve; SUP, suprascapular nerve; THL, long thoracic nerve; ULN, ulnar nerve.

^a^ Relating to MED and IOA.

While mechanical and other immunological trigger factors were found at a similar frequency, hepatitis E IgM antibodies, a marker of (sub)acute infection, were detected significantly more frequently in the subgroup with NAFL (46.7% versus 14.0%; *p* = 0.019).

**FIGURE 3:**
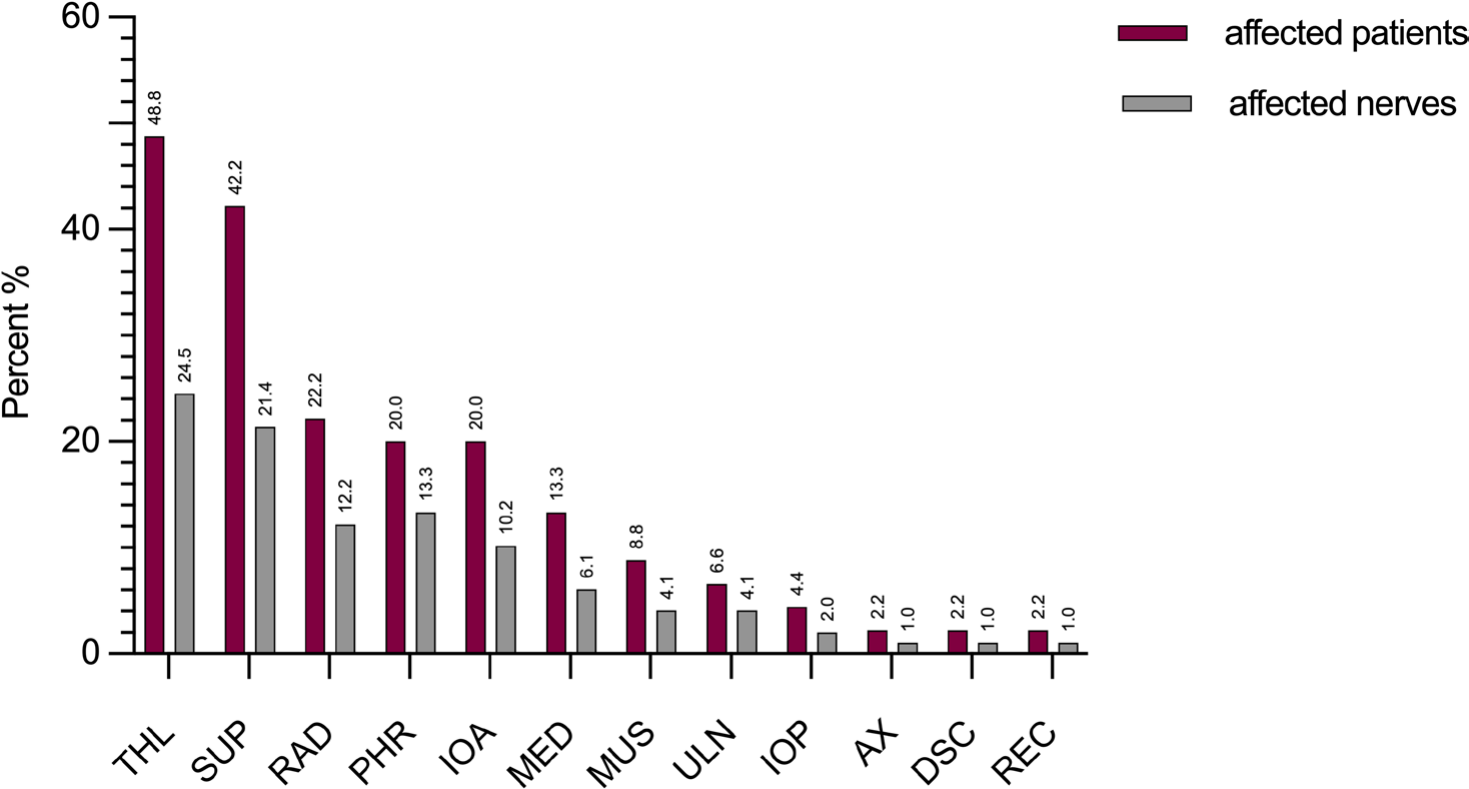
Frequency of involvement of individual nerves based on patients affected by NA and nerves affected by NA; patients referred directly via the diaphragm clinic were excluded. THL = long thoracic nerve; SUP = suprascapular nerve; PHR = phrenic nerve; IOA = anterior interosseous nerve; MED = median nerve; MUS = musculocutaneous nerve; ULN = ulnar nerve; IOP = posterior interosseous nerve; AX = axillar nerve; DSC = dorsal scapular nerve; REC = recurrent nerve.

Figure 3 illustrates the frequency with which the individual nerves were affected, both clinically and electromyographically. Thirty‐one of the 77 patients were initially referred to our diaphragm clinic due to diaphragmatic paralysis. These patients were excluded from Figure 3 to avoid bias towards the involvement of the phrenic nerve. The three most commonly affected nerves were the long thoracic nerve, the suprascapular nerve, and the radial nerve. These were followed by the phrenic nerve and the anterior interosseous portion of the median nerve.

While no clear preference for one side was observed in the majority of nerves, this was not the case for the long thoracic nerve, which was affected on the right side in 79% of cases and 4/5 NAFL were also found on the right side (Table 2).

**FIGURE 4:**
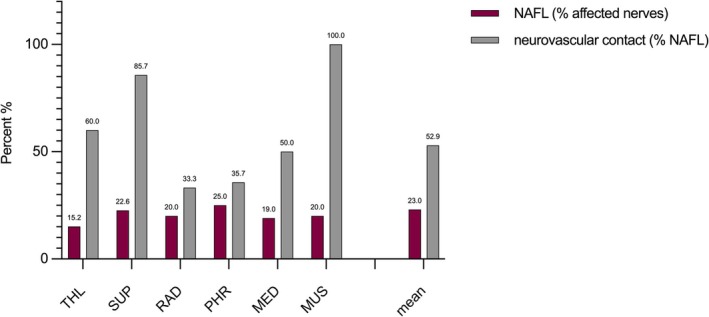
Prevalence of NAFL and neurovascular contacts of the affected nerves. THL = long thoracic nerve; SUP = suprascapular nerve; RAD = radial nerve; PHR = phrenic nerve; MED = median nerve; MUS = musculocutaneous nerve.

Overall, 31% of all patients and 20% of all affected nerves were found to have NAFL using sonography, with the phrenic nerve being the most commonly affected at 25%. In this cohort, at least one neurovascular contact in the NAFL area was identified in 63% (15/24) of patients and in 53% (18/34) of affected nerves, with 6/7 cases most frequently occurring in the suprascapular nerve (Figure 4). While a majority of patients with confirmed NAFL but without neurovascular contact reported a previous mechanical trigger, this was only observed in a smaller percentage of patients with such contact. However, the difference was not statistically significant (71.4 vs. 28.6%; *p* = 0.110).

## 4 Discussion

For the first time, this study focused on the prevalence of neurovascular contacts in the immediate vicinity of NA‐associated focal nerve lesions (NAFL).

The mean age of onset and the predominance of males in our cohort were consistent with data from earlier studies [4, 13, 14]. Excluding patients who were primarily referred to our diaphragmatic clinic, the frequency with which individual nerves were affected largely corresponded to existing data [4, 14]. Nevertheless, the phrenic nerve was involved in 20% of patients in our study, thus ranking it as the fourth most frequently affected nerve. In previous studies, this phenomenon was only observed in a range of 0% to 7.6% of patients with sporadic NA [14, 15, 16, 17]. One possible explanation for this phenomenon is that all patients in our study group were routinely asked about new dyspnea symptoms, and most of the cohort underwent diaphragmatic ultrasound. Compared with conventional chest X‐ray, this examination method has been demonstrated to exhibit superior sensitivity and, most notably, specificity in the diagnosis of diaphragmatic dysfunction (93/100% versus 90/44% in cases of unilateral diaphragmatic paralysis) [18, 19]. Conventional X‐ray diagnostics are inadequate, particularly for the diagnosis of bilateral diaphragmatic paralysis. Given the paucity of prior studies examining diaphragmatic sonography in patients with this condition, it is reasonable to hypothesize that the involvement of the phrenic nerve was frequently underdiagnosed.

The prevalence of NAFL in our cohort is consistent with previously published data, provided that ultrasound was used as the imaging modality [20]. A number of studies that employ MR neurography as a methodological approach have documented prevalences that are considerably higher. This discrepancy can be attributed, on the one hand, to the greater penetration depth of MRI and, on the other hand, to its lower spatial resolution compared to ultrasound, which results in different criteria for an MRI‐positive result. In one of the largest of these studies, a focal reduction in nerve diameter was deemed a sufficient criterion, obviating the necessity for a proximal cross‐sectional enlargement of the nerve [21]. In 53% of the NAFL detected by sonography in our study, direct contact with an artery crossing the nerve was demonstrated. For example, the transverse cervical artery was found to be in close proximity to those of the phrenic nerve, whereas the inferior ulnar collateral artery was found to be adjacent to those of the median nerve.

Assuming that these neurovascular contacts play a role in the pathophysiology of neuralgic amyotrophy, particularly in the development of NAFL, both the rarer occurrence of preceding mechanical trigger factors in patients with these contacts and the above‐mentioned discrepancies with the previous model could be conclusively explained.

**FIGURE 5:**
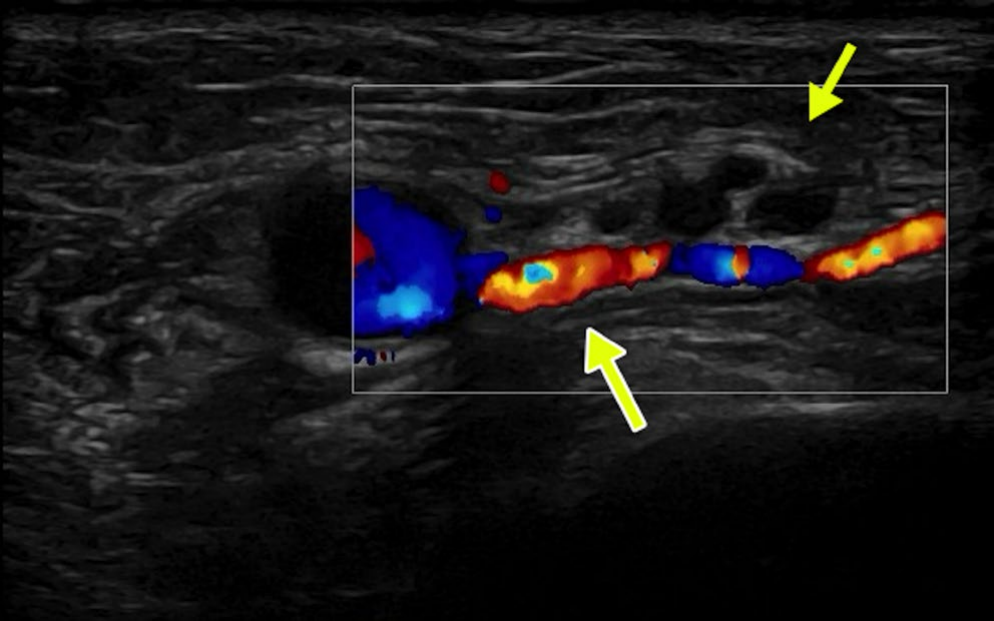
The median nerve (small arrow) and the dorsomedially located inferior ulnar collateral artery (large arrow).

Similar to traction on the nerve, it is conceivable that such contact can cause local disruption of the blood–nerve barrier due to arterial transverse and longitudinal pulsations. Such a pathomechanism has also been observed in the development of trigeminal neuralgia and other diseases [22]. Furthermore, an artery that runs transversely to the nerve may act as a fulcrum, thereby promoting the development of torsion. These mechanisms could explain the lack of preference for the dominant arm, as well as certain anatomical predilection sites that often correspond to locations of physiological neurovascular contacts. For instance, constrictions and Torsions of the median nerve are almost always found in the distal upper arm (on average, 33 ± 26 mm proximal to the medial epicondyle) during surgery or imaging procedures, rather than in the elbow area, where there is the greatest amount of mechanical stress from movement [23, 24, 25, 26, 27]. An anatomical study of adult cadavers revealed that, on average, the intersection of the inferior ulnar collateral artery and the median nerve is located 45 ± 11 mm proximal to the epicondyle [28]. Thus, the majority of NAFL occur between the intersection's minimum and maximum (27–63 mm). These findings suggest a correlation that determines the location of NAFL. This hypothesis also provides a convincing explanation for the preference of certain areas within the nerve cross‐section. The inferior ulnar collateral artery, for instance, generally courses dorsomedial to the median nerve. This could explain why, in most cases, the fascicles located there are affected, from which the anterior interosseous nerve forms further distally due to the somatotopic arrangement (Figure 5).

The significance of the extent of inflammatory activity is further substantiated by the observation that serological markers of (sub)acute hepatitis E infection were found to occur significantly more frequently in the subgroup of patients with NAFL (46.7% versus 14.0%; *p* = 0.019). Hepatitis E virus (HEV) has been recognized as a causative agent of NA, with both direct neurotropic effects and immune‐mediated mechanisms being discussed in the literature [29]. Cases of NA triggered by hepatitis E infection are characterized by a distinct clinical phenotype, with bilateral involvement patterns being significantly more common, as is involvement of the phrenic nerve or nerves of the lumbosacral plexus [30]. Together with our data, these findings indicate that the hepatitis E virus provokes a more robust (auto)immunological reaction than other pathogens do. A likely reason for this is the ability of HEV to cross the blood–brain barrier (and presumably also the blood–nerve barrier), replicate in neuronal cells, and trigger a local inflammatory response, as evidenced by experimental models and detection of HEV RNA in cerebrospinal fluid and nerve tissue [31].

The two main limitations of this study are, first, the small number of cases and, second, the retrospective nature of the data collection. However, given that the majority of the examinations were conducted in B‐mode and lacked color‐coded duplex sonography, it is plausible that neurovascular contacts can be identified more frequently in a prospective study design. Further studies are necessary to reproduce our data not only sonographically but also intraoperatively and to determine whether this will have an impact on future surgical strategies for nerve torsion.

## Author Contributions

J.F.H. and M.Z. contributed to the conception and design of the study; J.F.H. and A.L. contributed to the acquisition and analysis of data; J.F.H., V.L., W.W., and M.Z. contributed to drafting the text or preparing the figures.

## Funding

The authors have nothing to report.

## Conflicts of Interest

The authors declare no conflicts of interest.

## Data Availability

The data on which the results of this study are based are openly available in “Zenodo.” https://doi.org/10.5281/zenodo.18414901.
